# Developing a Technical-Oriented Taxonomy to Define Archetypes of Conversational Agents in Health Care: Literature Review and Cluster Analysis

**DOI:** 10.2196/41583

**Published:** 2023-01-30

**Authors:** Kerstin Denecke, Richard May

**Affiliations:** 1 Bern University of Applied Sciences Biel Switzerland; 2 Harz University of Applied Sciences Wernigerode Germany

**Keywords:** mobile phone, user-computer interface, telemedicine, communication, delivery of health care and methods, delivery of health care and trends

## Abstract

**Background:**

The evolution of artificial intelligence and natural language processing generates new opportunities for conversational agents (CAs) that communicate and interact with individuals. In the health domain, CAs became popular as they allow for simulating the real-life experience in a health care setting, which is the conversation with a physician. However, it is still unclear which technical archetypes of health CAs can be distinguished. Such technical archetypes are required, among other things, for harmonizing evaluation metrics or describing the landscape of health CAs.

**Objective:**

The objective of this work was to develop a technical-oriented taxonomy for health CAs and characterize archetypes of health CAs based on their technical characteristics.

**Methods:**

We developed a taxonomy of technical characteristics for health CAs based on scientific literature and empirical data and by applying a taxonomy development framework. To demonstrate the applicability of the taxonomy, we analyzed the landscape of health CAs of the last years based on a literature review. To form technical design archetypes of health CAs, we applied a k-means clustering method.

**Results:**

Our taxonomy comprises 18 unique dimensions corresponding to 4 perspectives of technical characteristics (setting, data processing, interaction, and agent appearance). Each dimension consists of 2 to 5 characteristics. The taxonomy was validated based on 173 unique health CAs that were identified out of 1671 initially retrieved publications. The 173 CAs were clustered into 4 distinctive archetypes: a text-based ad hoc supporter; a multilingual, hybrid ad hoc supporter; a hybrid, single-language temporary advisor; and, finally, an embodied temporary advisor, rule based with hybrid input and output options.

**Conclusions:**

From the cluster analysis, we learned that the time dimension is important from a technical perspective to distinguish health CA archetypes. Moreover, we were able to identify additional distinctive, dominant characteristics that are relevant when evaluating health-related CAs (eg, input and output options or the complexity of the CA personality). Our archetypes reflect the current landscape of health CAs, which is characterized by rule based, simple systems in terms of CA personality and interaction. With an increase in research interest in this field, we expect that more complex systems will arise. The archetype-building process should be repeated after some time to check whether new design archetypes emerge.

## Introduction

### Background

In recent years, artificial intelligence (AI) has become increasingly successful because of its powerful capabilities and possibilities for a variety of application areas. Precisely, AI is the use of computers and technology to simulate intelligent behavior and critical thinking comparable with a human being [1]. The possibility of machines behaving in such a way was originally raised by Alan Turing and further explored starting in the 1950s. However, the evolution of AI and natural language processing generates new opportunities for conversational agents (CAs) that communicate and interact with individuals. Enabling those agents to speak becomes straightforward through current speech-to-text and text-to-speech technology.

### Motivation

In this work, we considered CAs as dialog systems interacting with users in a humanlike manner. Chatbots are a subset of CAs as they are also dialog systems, but the user interface is often button focused. In the health domain, CAs became popular as they allow for simulating the real-life experience in a health care setting, which is the conversation with a physician. They have been tested for patient education [2], supporting behavior change [3], or delivering cognitive behavioral therapy [4]. Furthermore, hospitals and private clinics use CAs to triage and clerk patients even before they come into the consulting room. The intelligent agents ask relevant questions about the patients’ symptoms, with automated responses that aim to produce a comprehensive medical history for the physician [5]. In combination with regular care provision, CAs offer the great opportunity of improving the efficiency of patient care delivery, simplifying processes, and ensuring quality of care.

However, to develop and assess CAs, it is initially important to know how CAs are designed, especially from a technical perspective. Thus, the objective of this work was to develop a taxonomy of technical aspects characterizing health CAs. This taxonomy was used to form unique archetypes. Each of these archetypes was expected to be different in terms of its technical properties. The archetypes aimed to deliver insights into the current landscape of health CAs. Moreover, they could form the basis to specify sets of relevant evaluation metrics for each archetype to ensure harmonized quality assessment.

Health CAs differ from general domain CAs in multiple aspects. The conversation content has to be tailored based on the application area, use case, and user context, and has to address privacy [6]. Similar to the physician-patient communication along a treatment process, health CAs can require multiple interactions over a period. Interaction frequency can be multiple times a day. These interactions require information to be retained persistently between sessions, and sometimes it is even necessary to refer to information from previous dialogs. As patient knowledge increases over time, the language and content should adapt to different knowledge levels. A crucial aspect within health care communication is empathy; thus, a health CA must also “address social, emotional and relational issues” [6]. These peculiarities of health CAs lead to technical characteristics that can be used to characterize health CAs.

### Related Work

There are some classification frameworks for health CAs. The approaches are dispersed into different thematic axes such as application area. Janssen et al [7] suggested a taxonomy for CAs focusing on general CA design, the technological structure, and the context in which the CA is embedded. Information on data processing is not included in this taxonomy. Trofymenko et al [8] presented a classification of CAs for the purpose of “a clear understanding of nature, approaches to creation, advantages and disadvantages” of CAs. Their classification distinguished 7 dimensions: purpose, location, interface, number of users, form of access, algorithm, and functionality. This taxonomy focuses on the use case perspective but misses information on the technical realization. Nissen et al [9] introduced a design taxonomy to characterize user-chatbot relationships. Their taxonomy focuses on the time horizon of a CA and not on technical characteristics. They distinguished 3 time-dependent archetypes of open-domain CAs: ad hoc supporters, persistent companions, and temporary advisors. Other taxonomies considered specific aspects of CAs. As exemplified, Welivita and Pu [10] introduced a taxonomy of empathic chatbot responses. Feine et al [11] built a taxonomy of social cues of CAs concentrating on verbal, visual, auditory, and invisible aspects. None of the available taxonomies focuses on technical characteristics specifically considered for health CAs.

In addition, there are reviews of health CAs and descriptions of specific characteristics without any systematic consolidation of the findings in terms of a taxonomy. For instance, Vaidyam et al [12] reviewed chatbots and their particularities in mental health. Tudor Car et al [13] conducted a scoping review on the characteristics of general health CAs. For these and similar reviews related to health CAs, extraction tables were defined. They reflect the focus of the review but are insufficient as taxonomies of technical characteristics.

Other CA research identified archetypes of CAs from different perspectives. Diederich et al [14] distinguished CA design platforms. They identified text-based, domain-specific CA platforms; general-purpose, cloud-based CA platforms; and multilanguage, integrative CA platforms. Furthermore, they grouped CAs based on their design and interaction paradigms [15]. More specifically, they considered 4 dimensions affecting interaction: context, human, perception, and outcome. However, they did not concentrate on a particular domain as we did by focusing on health CAs. Bahja and Lowry [16] formed archetypes from a customer-centric value chain perspective. They specified 4 archetypes for service-oriented CAs: informer, planner, facilitator, and performer. Jovanović et al [17] formed archetypes regarding health provisioning roles (diagnostic, prevention, or therapy).

Overall, the related work we identified lacks a comparable taxonomy and archetypes focusing on the technical implementation aspects of health CAs. Although there is already some research available trying to enlighten the facets and appearances of CAs, a taxonomy focusing on the technical aspects of health CAs is still missing. We fill this gap by suggesting a taxonomy of technical CA characteristics. The questions driving our study are as follows: (1) What are the technical characteristics of health CAs? (2) Which archetypes of health CAs can be empirically identified using technical characteristics? (3) Which distinctive properties exist to characterize single groups of health CAs?

**Figure 1:**
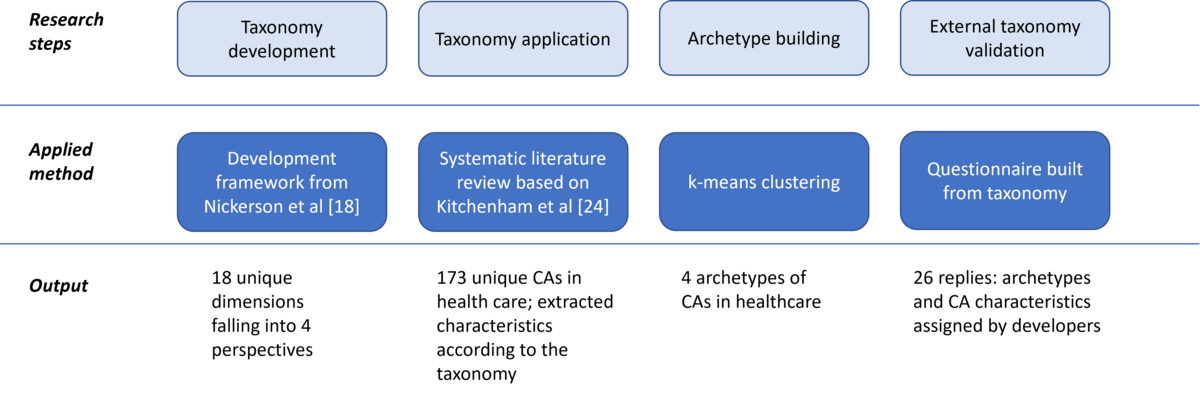
Overview of all research steps, applied research methods, and outputs of the methods [18,24]. CA: conversational agent.

To answer these questions, we developed a technical-oriented taxonomy for health CAs based on scientific literature and empirical data. To develop the taxonomy, we analyzed the landscape of health CAs of the last 12 years by conducting a literature review. To form technical archetypes of health CAs, we deployed a cluster analysis and identified 4 CA archetypes. All these steps are shown in Figure 1 [18,19]. Finally, we outlined practical implications and recommendations and discussed the approach.

The contributions of this work are to (1) present a technical-oriented taxonomy for describing basic characteristics of health CAs, which will allow researchers in the field to classify their health CAs and compare them with others, which—once applied as a reporting guideline for health CAs—will allow users of health CAs to inform on important aspects related to a health CA, in particular on data processing, which will contribute to perceived safety; (2) provide an up-to-date review of health CAs; (3) identify design archetypes of health CAs required to define specific evaluation metrics for different archetypes; and (4) identify the main challenges and research directions for future work.

## Methods

### Taxonomy Development Procedure

In this study, a taxonomy of technical characteristics for health CAs was developed based on scientific literature and empirical data. In this way, we pursued the goal of providing a systematic representation of technical characteristics of health CAs and developing a better understanding of technical elements of health CA design. Thus, our taxonomy also allowed for characterizing the current stage of the technology included in health CAs.

The taxonomy development is an adaptation of the 7-step framework for taxonomy development proposed by Nickerson et al [18]. Textbox 1 lists these steps together with our adaptions. More details are given in the following sections.

In a first step, we formulated the meta-characteristic, which is “the most comprehensive characteristic that will serve as the basis for the choice of characteristics in the taxonomy” [18]. We defined the meta-characteristics as the technical characteristics of health CAs. We considered *technical characteristics* as distinctive technical features that frame the CA capabilities in terms of human interaction and conversation.

The second step comprised the determination of ending conditions to finalize the iterative taxonomy development process. We considered 5 subjective ending conditions suggested by Nickerson et al [18] as our ending conditions (ie, the taxonomy should be concise, robust, comprehensive, extendable, and explanatory). As we did not apply the taxonomy to concrete health CA implementations, the other suggested ending conditions were irrelevant in our process. In the third step, we decided on the taxonomy development approach, which we determined to be conceptual to empirical. Therefore, in the fourth step, we abstracted a preliminary conceptual taxonomic structure based on the CA development experiences of the authors [2,20-22] and existing research on health CAs [9,14,23,24]. This taxonomy was discussed among all authors. In addition, it was discussed with 3 researchers working on health CAs. All characteristics of a dimension were considered as exclusive (ie, for a CA, only 1 characteristic can be true within 1 dimension). To assess whether the ending conditions were met, we used the taxonomy developed based on our experiences and applied it to health CA retrieved by the literature review (see *Taxonomy Application* section). When necessary, we included additional characteristics. The resulting taxonomy fulfilled the specified ending conditions.

**The 7 steps for taxonomy development proposed by Nickerson et al [18] together with our adaptations.**

- The 7 steps by Nickerson et al [18]
  Determine meta-characteristics
  Determine ending conditions (Nickerson et al [18] suggest 7 subjective and 8 objective ending conditions)
  Decide on approach (Nickerson et al [18] suggest 1 of 2 options: empirical to conceptual or conceptual to empirical)
  Conceptualize (new) characteristics and dimensions of objects
  Examine objects for these characteristics and dimensions
  Create (revised) taxonomy
  Check whether ending conditions are met
- Adaptation in our work
  The technical characteristics of health conversational agents (CAs) are considered meta-characteristics.
  We consider 5 subjective ending conditions from the suggestions by Nickerson et al [18]: taxonomy should be concise, robust, comprehensive, extendable, and explanatory.
  We follow the approach conceptual to empirical.
  A preliminary conceptual taxonomic structure is abstracted based on the authors’ health CA development experiences.
  Characteristics and dimensions are added to the taxonomic structure based on the authors’ health CA development experiences.
  Revision of the taxonomy is based on input from discussions with 3 experts.
  The taxonomy is applied to the health CAs included in the review; additional characteristics are added when needed.

### Taxonomy Application

To identify health CAs, we used a literature review following the guidelines by Kitchenham et al [19]. We decided on a literature review for the following reasons: (1) identifying CAs in app stores is biased because of country restrictions (not all CAs are available in the app stores of a particular country); (2) we wanted to consider CAs of a certain quality, which can be expected when using literature databases that only include peer-reviewed publications; and (3) we needed technical implementation details that might be missing in descriptions of CAs in app stores. We searched for relevant scientific papers on PubMed, ACM Digital Library, and IEEE Xplore published between 2010 and 2022 and written in English. To identify appropriate literature, we defined the following search string:

(application OR app OR approach OR implementation) AND (chatbot OR bot OR conversation OR conversational user interface) AND (health OR healthcare) 

As PubMed only lists publications from the medical domain, we excluded the terms “health OR healthcare” when searching PubMed. Only publications that were peer-reviewed conference papers or journal articles of original work were included. Furthermore, the publication had to present a concrete CA applied in health care. We excluded papers not dealing with a concrete health care–related CA or only describing the design process, as well as reviews and meta-analyses.

**Figure 2:**
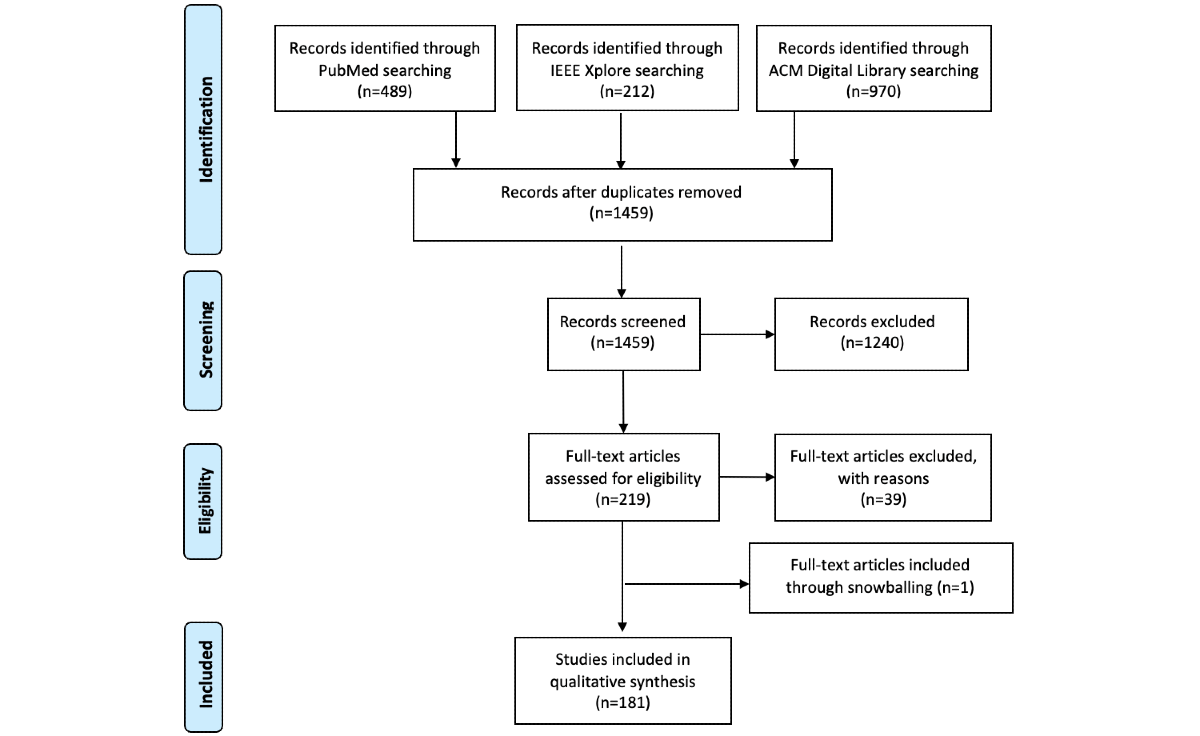
Flowchart of the literature review.

The search was conducted on April 4 and 5, 2022, resulting in 212 papers from IEEE Xplore, 970 papers from ACM Digital Library, and 489 papers from PubMed (see the flowchart in Figure 2). After a first exclusion process, 13.11% (219/1671) of the retrieved items were assessed for eligibility by examining titles and abstracts, where each reviewer looked at half of the papers. In a second round, the full texts were carefully considered by the reviewers to confirm eligibility. Overall, we assessed 82.6% (181/219) of the papers and extracted the characteristics based on our taxonomy. When necessary, characteristics were added to the taxonomy. In more detail, 2 researchers went through the list of relevant CAs (split into 2 parts; each researcher assessed 1 part). Each CA was analyzed based on the descriptions in the related paper. Data reflecting information from our taxonomy were extracted and filled in a standardized Microsoft Excel (Microsoft Corp) spreadsheet.

### Archetype Building

To form unique archetypes, we implemented k-means (via scikit-learn in Python [version 3.9; Python Software Foundation]) as an unsupervised clustering algorithm to automatically recognize patterns in our extracted features [25] based on their technical characteristics. First, the text values (ie, characteristics of the taxonomy) of 13 selected taxonomy dimensions were converted into representative numerical values. This step was required as the clustering algorithm required numerical data and our characteristics were textual. To mitigate the threat of affecting our results’ external validity, we excluded values of 4 out of 18 dimensions (ie, data privacy, data exchange, hosting, and internet access) because of a lack of information. For most cases (ie, data privacy, 157/173, 90.8%; data exchange, 120/173, 69.3%; hosting, 83/173, 48%; internet access 56/173, 32.4%; and sentiment analysis), we were unable to extract corresponding information on these categories from the papers (see the following section). In addition, we excluded the dimension sentiment analysis as it was recognized as insufficiently discriminative for archetype building; for 83.5% (145/173), the value was neglecting integration of sentiment analysis. The conversion was supervised by the second author, who also manually fixed incorrect assignments. Second, cluster seeds were initialized randomly according to a defined number of expected clusters. To generate an appropriate number of clusters while maximizing the distance between clusters, we used the elbow method [26], suggesting 4 clusters as appropriate to our data set. Third, the Euclidean distance between each point and seed was calculated (ie, the smallest distance became part of the given seed). Fourth, after covering the data, the seeds were placed in the centroids of the clusters, becoming their representatives. Fifth, the numerical values were converted to their text values, again supervised and manually fixed by the second author.

## Results

### Overview

Our taxonomy comprises 18 unique dimensions corresponding to 4 perspectives of technical characteristics (Table 1): agent appearance, domain, interaction, and data processing. Each dimension consists of 2 to 5 characteristics. The dimensions cover aspects related to the CA personality and its embodiment as well as several technical aspects such as hosting, internet access, privacy, application technology, or use of sentiment analysis methods. The complete data extraction table is available in Multimedia Appendix 1.

**Table 1:** Final taxonomy of technical characteristics of health conversational agents (CAs) with 18 dimensions and corresponding characteristics; when applicable, the references indicate from which publications the characteristics were adapted.

Level 1 (perspective) and level 2 (dimension)		Level 3 (characteristic)
**Agent appearance**		
	Personality of CA	Simple Complex
	Embodiment [27]	None Avatar Physical
	Application technology	Virtual reality Augmented reality Vocal Normal
	Intelligence framework [14,24]	Rule based Self-learning
	Sentiment or emotion detection [14]	Yes No
**Setting**		
	Context [14,23]	General-purpose Domain-specific
	Service duration [9,24]	Ad hoc supporters Persistent companions Temporary advisors
	Human involvement	Dyad Triad Quadriad
**Interaction**		
	Input mode and output mode [14,23]	Written Spoken Visual Hybrid Haptic
	Service channel [14,23]	Smartphone-embedded Software Social media Website (web-based) Smart speaker
	Device	PC Mobile device Both Other
	Language [14]	Single language Multilanguage
	Integration mode	Stand-alone Part of a system
**Data processing**		
	Internet access	On the web Offline
	Hosting [14]	Local Outsourced Other
	Data exchange with third-party device or service	Access Storing Both None
	Data privacy	Privacy policy Data encryption Both Nothing

### Agent Appearance

Considering a CA as a subject, we refer by agent appearance to the visual appearance but also to the intelligence or personality of a CA. The agent appearance can be categorized in 5 dimensions. The *personality of the CA* indicates the human likeness of a CA. The *embodiment* illustrates whether the CA is embodied (eg, by an avatar or even a robot). The *application technology* refers to the technological environment in which the CA is embedded. The *intelligence framework* indicates whether the CA is primarily based on rules or machine learning is applied [4,28]. The dimension *sentiment or emotion detection* characterizes whether the CA can automatically detect users’ emotion or sentiment during an interaction [28]. Sentiment analysis would be a prerequisite for being able to generate empathic responses to a particular user.

### Setting

The setting perspective comprises 3 dimensions and characterizes how a CA is embedded in the environment of a user. The *context* illustrates whether the CA serves a specific domain or can interact on any topic with its users [2,3,28]. *Service duration* [4,29] refers to the services offered by a CA. It can be either short-term or cover a longer period and be designed for solving a concrete, time-limited task or focus on a longer interaction. Ad hoc supporters are designed for a short, isolated, one-time–only interaction. Persistent companions are long-term CAs designed for longer, interdependent, and perpetual interactions. Conversation can develop into new directions proactively. Temporary advisors are medium-term CAs [4,29]. *Human involvement* denotes whether the interaction is between a user and the agent or whether other individuals are involved in the human-agent interaction.

### Interaction

The interaction perspective describes a CA in terms of the human-computer interaction. It comprises 5 dimensions. *Input or output mode* refers to the primary way in which a CA can interact with the user [14,23]. *Service channel* characterizes where the CA is primarily embedded [4,28]. A CA is deployed on a particular device; thus, we included the dimension *device*. Relying on language, a CA might support multiple languages or only one [28]. This is expressed in the dimension *language*. The dimension *integration mode* indicates whether the CA is part of a system or works as a stand-alone application.

### Data Processing

The fourth perspective refers to the processing of data by the CA. The dimension *internet access* indicates whether the CA works in web-based or offline mode (ie, requires internet access). *Hosting* characterizes the data hosting type used by the CA [28]. CAs can exploit different services such as speech-to-text or text-to-speech services. They can also exchange data with other devices. The dimension *data exchange with third-party services or devices* denotes whether the CA stores or accesses data from or to third-party services or devices (eg, wearables or electronic health records). *Data privacy* refers to privacy aspects of the personal health data collected, stored, or processed by the CA.

### Application of the Taxonomy

#### Overview

**Figure 3:**
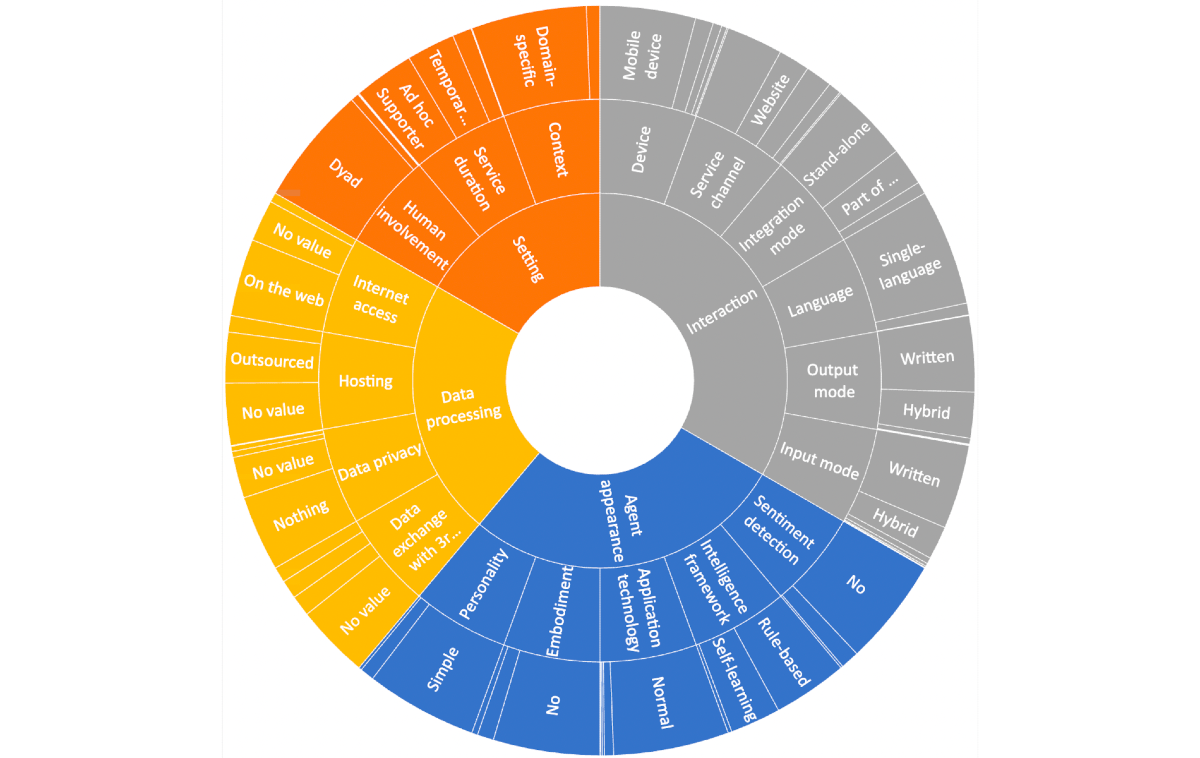
Mapping of the conversational agents assessed to our taxonomy (N=173). The size of the outer fields corresponds to the number of agents with these features.

In this section, we summarize the results when applying the taxonomy to the 181 health CAs that were identified in the literature review. Figure 3 provides an overview of the characteristics of the health CAs. More details are provided in the next section.

#### Analyzed CAs

##### Included Studies

**Figure 4:**
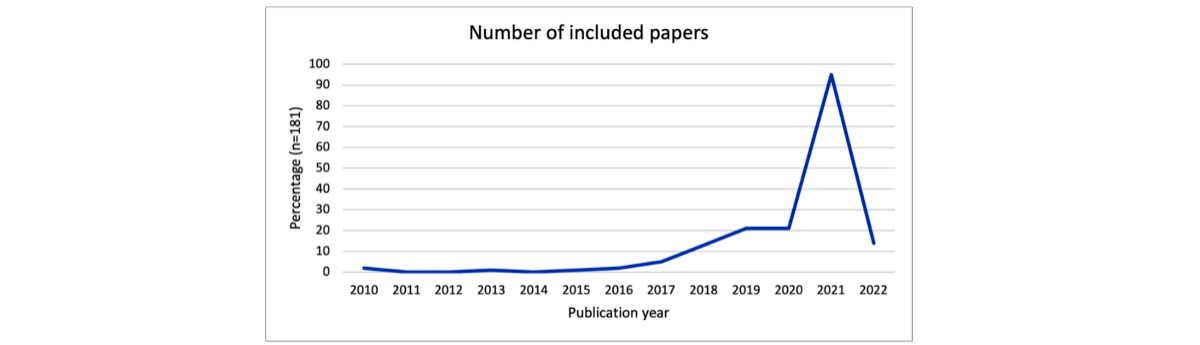
Distribution of the included publications on health conversational agents over the years.

Figure 4 shows the distribution of the publication years of the 181 papers identified through the literature review. It can be seen that more papers were published since 2018. In 2011, 2012, and 2014, no papers were identified, and we collected 1.1% (2/181) of the papers from 2010 and 2016 and 2.8% (5/181) from 2017. The number of publications increased suddenly in 2021 from 21 to 96. We note that the drop in 2022 results from the search date, which was in April 2022. Accordingly, we are aware that 2022 cannot be included in the trend analysis of the increased number of publications (compared with 2021).

Of the 181 papers from which data were extracted, we identified 173 (95.6%) unique CAs as, for some CAs, more than one paper was included in the review. The results including the references of the considered CAs will be published as a replication package. With these 173 systems, we demonstrated the application of the taxonomy and characterized the landscape of health CAs from a technical perspective. In the following sections, we describe the characteristics of the assessed CAs according to our taxonomy.

##### Agent Appearance

In the *intelligence framework* dimension, which corresponds to the *agent appearance* perspective, most (100/173, 57.8%) of the CAs investigated tended to function based on rules, and 39.3% (68/173) applied machine learning. For 2.9% (5/173), no information could be identified. Consistently with this, sentiment analysis was not integrated in most of the CAs (145/173, 83.8%). Only 14.5% (25/173) used sentiment analysis techniques (there was no information for 3/173, 1.7%). In the *personality* dimension, we recognized that 86.7% (150/173) of the CAs were implemented with a simple personality, and 11% (19/173) were implemented with a complex personality. For 2.3% (4/173), information on the CA personality was unavailable. Most (143/173, 82.7%) CAs were implemented without embodiment, 12.7% (22/173) were implemented as avatars, and 4.6% (8/173) were realized with physical embodiment of the agent. In the *application technology* dimension, most (155/173, 89.5%) CAs applied normal technologies (ie, text-based or standard graphical user interface); 6.9% (12/173) applied virtual reality (VR); 0.6% (1/173) applied augmented reality; 1.2% (2/173 applied vocal technologies; and for 1.7% (3/173) of the CAs, we could not identify the application technology.

##### Setting

In terms of the *context* dimension, most (155/173, 89.6%) CAs were clearly domain specific. A total of 10.4% (18/173) were general-purpose CAs. The service duration was short-term or medium-term—we identified ad hoc supporters (81/173, 46.8%), persistent companions (26/173, 15%), and temporary advisors (65/173, 37.6%); no decision was possible for 0.6% (1/173) of the CAs. Human involvement mainly focused on a single user who interacts with the CA (160/173, 92.5%). In total, 4.6% (8/173) of the CAs involved a clinician [30-35]. Either the clinician was enabled to access the conversation protocol or they were able to interact with the patient through the chat interface. In total, 1.2% (2/173) of the systems were designed for children. In the latter systems, parents and physicians were enabled to join the system and review the conversation between the children and CAs or interact with the children [36,37]. Another set of systems (5/173, 2.9%) enabled conversations between the CA and multiple users, whether communities of patients or several clinicians [38].

##### Interaction

A total of 90% (156/173) of the CAs were only able to talk and understand a single language. Most systems relied on written input (113/173, 65.5%) and output (102/173, 58.8%). Several options (ie, a hybrid interaction mode for input and output) were reported for 25% (45/173; input) and 35% (61/173; output) of the CAs. A total of 4.6% (8/173) of the CAs only used speech as input and output. Visual interaction was reported for 2.3% (4/173) of the CAs as input and 0.6% (1/173) of the CAs as output. In total, 0.6% (1/173) of the CAs allowed for haptic input [39].

In terms of the *service channel* dimension, CAs were made available mainly through smartphones (76/173, 43.9%), were integrated into social media messengers (36/173, 20.8%), or were accessible through websites (42/173, 24.3%). A minority of CAs were available through smart speakers (2/173, 1.2%; [40]); no corresponding information was available for 9.8% (17/173). Most (106/173, 61.3%) were implemented as stand-alone systems; 29% (51/173) were part of a system; and for 9.2% (16/173), no information regarding the *integration mode* dimension was available. The most prominent device through which CAs could be accessed were mobile devices (128/173, 74%), 13.9% (24/173) of the systems were accessible through a PC, and 4% (7/173) were accessible through other devices. In total, 1.2% (2/173) of CAs accessible through PC and mobile devices were identified. Information was missing for 6.9% (12/173) of the CAs.

##### Data Processing

Information on data privacy, hosting, and data exchange with other systems was rarely described for the CAs assessed. In total, 90.1% (157/173) of the papers did not mention how data privacy was addressed. For 4.6% (8/173) of the CAs, a privacy policy was mentioned; 0.6% (1/181) of the papers reported on applied data encryption; and 3.9% (7/181) of the papers reported both a privacy policy and data encryption to be implemented in their CAs. In total, 60.1% (104/173) of the CAs ran on the web, thus requiring internet access; 7.5% (13/173) ran offline; and for 31.4% (56/173), we could not extract the corresponding information. Furthermore, information on data access and storing and the use of third-party devices and services was rare. For 69% (120/173) of the CAs, no information on this was available. A total of 14.5% (25/173) of the papers reported on access to third-party systems; 16% (28/173) reported on access and storage on third-party tools. Regarding the *hosting* dimension, for almost half (83/173, 48%) of the CAs, we could not identify related information; for 39% (68/173), we assumed that the CA hosting was outsourced given the information provided. Local hosting was identified for 12.7% (22/173) of the CAs.

### Technical Archetypes of CAs

**Figure 5:**
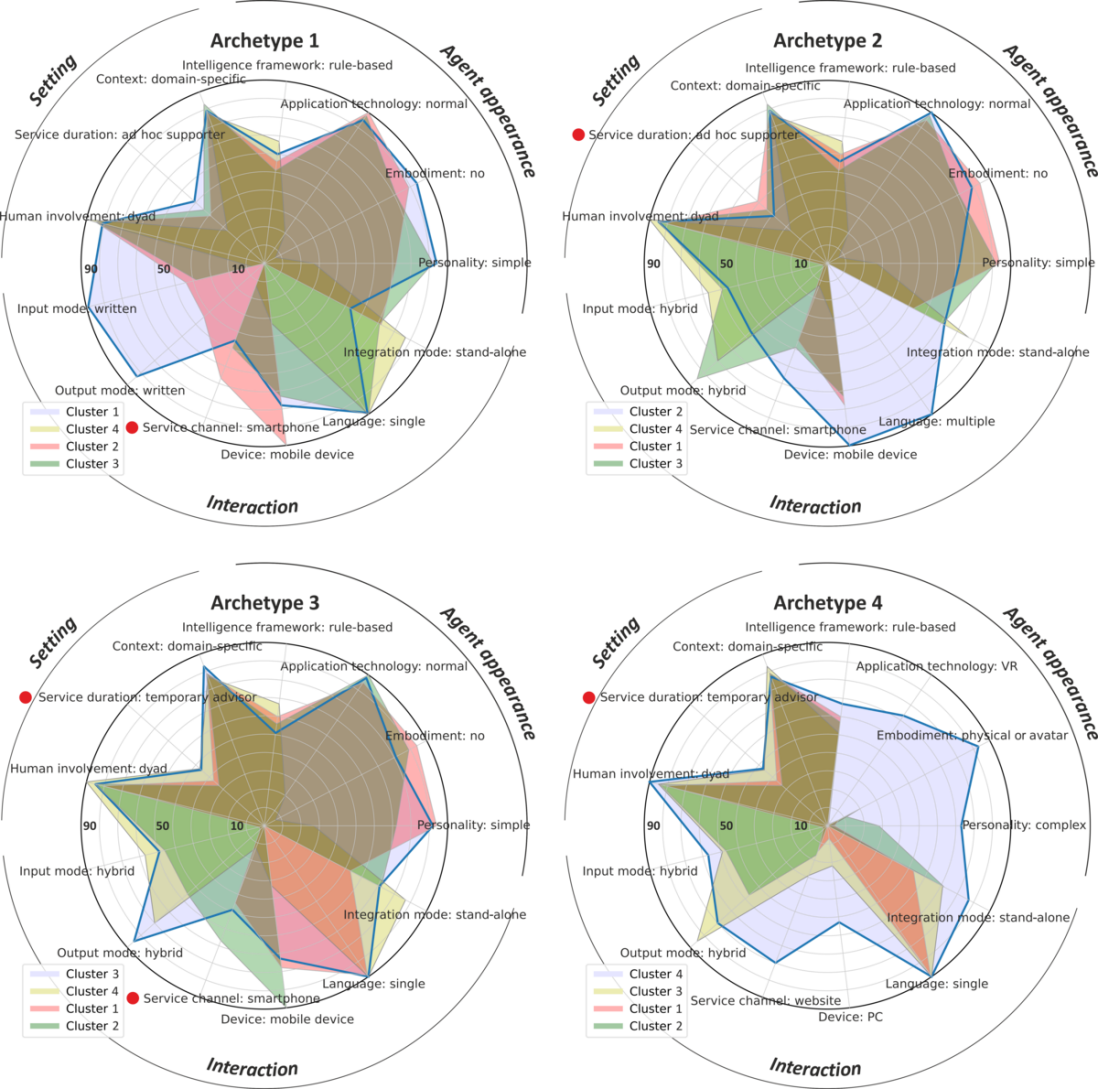
Radar charts of the 4 archetypes based on 4 clusters. Each chart shows the actual archetype characteristic fulfillment by all clusters (in percentages; the red points indicate nondominant characteristics), highlighting the dominant cluster the archetype is based on (blue line). VR: virtual reality.

The clustering process resulted in 4 distinctive archetypes of health CAs (Multimedia Appendix 2). We named the archetypes as follows: (1) text-based ad hoc supporter; (2) multilingual, hybrid ad hoc supporter; (3) hybrid, single-language temporary advisor; and (4) embodied temporary advisor. The names reflect the main characteristics and the service duration. They have in common that they are mainly rule based and domain specific. Moreover, they are often implemented as stand-alone systems, and no additional users to the user and agent interact with each other. Note that we excluded 5 categories from the clustering because of missing information, namely, all dimensions of the *data processing* perspective (data privacy, hosting, data exchange, and internet access) and 1 dimension of the *agent appearance* perspective (sentiment analysis). Only 14.4% (25/173) of the systems integrated sentiment analysis—therefore, this was also not a distinctive feature for clustering. The results of the clustering can be found in Multimedia Appendix 2 and Figure 5.

Archetypes 1 to 3 show a clear difference from archetype 4 in the 4 dimensions—*personality* (simple), *embodiment* (no), *application technology* (normal), and *device* (mobile device). The main difference of archetype 1 compared with the others is the service duration. A total of 50.5% (87/173) of the CAs assigned to this cluster were ad hoc supporters, and the input and output mode were written. For the other 3 archetypes, there was a larger variability in the service duration. Archetype 2 can be distinguished from the others via the multilingualism and a larger proportion of smartphone-embedded software as service channel. Archetype 4 differs from the others in the service channel, VR as application technology, and a complex personality. In Textbox 2, we summarize the characteristics per archetype.

**Archetype characteristics.**

- Archetype 1: text-based ad hoc supporter (eg, Chetlen et al [41])
  The first group (107/181, 59.1% of conversational agents [CAs]) contains CAs that interact with users mainly via text input and output in 1 language. The CA personality is simple, without any kind of embodiment. The knowledge is domain specific, and most systems are rule based, run on a mobile device, and are implemented as stand-alone software. The interaction time is rather short—most systems are ad hoc supporters.
- Archetype 2: multilingual, hybrid ad hoc supporter (eg, Horii et al [42])
  The second group (18/181, 9.9% of CAs) offers multiple input and output options and also covers multiple languages. Technical implementation is again rule based. The CA personality is simple, without embodiment. Running on a mobile device, it is mainly used as an ad hoc supporter. However, medium- and long-term service durations are also covered.
- Archetype 3: hybrid, single-language temporary advisor (eg, van Baal et al [43])
  The third group (41/181, 22.7% of CAs) contains CAs that provide multiple input and output options but, again, only in 1 language. The interaction time is medium (ie, these systems are often temporary advisors), but they are also used as ad hoc supporters.
- Archetype 4: embodied temporary advisor (eg, Tielman et al [44])
  The fourth group (15/181, 8.3% of CAs) of systems comprises CAs with complex personalities, with physical embodiments or avatars, and realized with virtual reality. In contrast to the other 3 archetypes, this group of CAs runs on websites, and a PC is often required. Data input and output is possible in multiple manners, but only 1 language is supported. Most of those systems have a medium-term use duration (ie, they are temporary advisors).

## Discussion

### Principal Findings

Our research resulted in a taxonomy of technical characteristics for health CAs consisting of 18 dimensions that are grouped into 4 perspectives: agent appearance, setting, interaction, and data processing. Existing health CAs can be grouped into 4 archetypes, each of which has specific characteristics. The 4 archetypes are distinguished from each other in the input and output modalities, service duration, or support for multiple languages. One group of systems is more complex in terms of CA personality, which we labeled *embodied temporary advisor*.

### Theoretical and Practical Implications

We developed a domain-specific taxonomy characterizing health CAs from a technical perspective. The taxonomy can be used as a reporting guideline by researchers and developers to describe essential details of their health CAs. This would ensure that details of the technical implementations are provided in a harmonized manner. From a research perspective, this would support the comparison of health CAs addressing the same use case. From a health CA user perspective, it ensures the transparency of the underlying technologies. It allows for the better judgment of the underlying technology and data processing of the health CA that a user is supposed to interact with. In particular, transparency related to data processing could contribute to perceived user safety. However, it would require technical knowledge to understand the characteristics. From a developer perspective, the application of the taxonomy allows for the classification of a system into one of the design archetypes. It would also help possibly associate technical implementations to outcomes and compare outcomes of different CAs relating to the technical criteria. As exemplified, questions such as the following—“For which use cases machine learning integrated in a health CA is useful”—could be answered.

Other researchers have so far focused on other aspects of health CAs, such as CA platforms [14] or the time dimension [9], or on a domain-independent taxonomy [7]. Some of our results are comparable with the results of other researchers. Janssen et al [7], for example, found that, in most cases, CAs (without specific focus on health care) are rule based, only 1 individual participant interacts with the CA, and the CAs are without embodiment. This also holds true for our results. However, in contrast to their results, health CAs in our study seemed to be distributed as smartphone-embedded software rather than through social media. We can explain this by the requirements regarding data privacy that have to be considered in health care applications. Nissen et al [9] focused in their archetypes on domain-specific, text-based chatbots and the time dimensions. We considered their results in our taxonomy by including the dimension *service duration*. Our archetypes also reflect the time dimension of the interaction [9].

In total, 3 out of 4 archetypes are quite similar (archetypes 1, 2, and 3). However, they differ in the length of interaction (ad hoc supporter vs temporary advisor), input and output options, and number of supported languages. Most (107/181, 59.1%) CAs were assigned to archetype 1 (simple, text-based ad hoc supporter). The second most frequently occurring systems fell into archetype 2, the simple, hybrid temporary advisor. The fourth archetype subsumes a different, unique group of systems that seem to be more complex. We argue that this archetype exemplifies the strong interconnection between the higher complexity of specific CA characteristics—such as personality, input and output options, embodiment, and application technology—and the device. In this context, it seems logical to implement an embodied avatar (possibly in combination with physical components) if the application is based on a complexly designed VR world, leading to more immersive effects for patients [2]. Moreover, this is why it is conceivable that the more complex the CA is (ie, realization of an embodied CA in a VR), the more complex the system requirements are (ie, a PC is needed instead of a smartphone because of its limited resources). However, such complex applications will probably also run on smartphones in the future because of their increasing capabilities.

Surprisingly, none of the archetypes represent systems based on machine learning (even though there were a few included in the literature review). CA systems are often labeled as “artificial intelligence,” but it turned out that most of the systems (100/173, 57.8%) were rule based with limited intelligence. A reason might be that self-learning systems are difficult to control and can result in unexpected behavior, which produces a risk for patient safety. Another reason might be that the use cases for which CAs are developed in health care do not produce a demand for sophisticated machine learning and can be realized much more easily using rules. Third, training data in terms of health care dialogs for the various use cases might be missing.

Regarding the fulfillment of the dominant archetype characteristics by archetype-related publications (Figure 4), we can see that the actual fulfillment was not dominant (ie, >50% fulfillment) for every characteristic. This finding leads to the fact that every archetype consists of a certain number of dominant or nondominant properties, giving every archetype a certain weight regarding specific characteristics (eg, complex personalities for archetype 4 but with a nondominant service duration). In addition, the publications referring to specific archetypes also fulfilled characteristics or combinations of specific characteristics of other archetypes, highlighting especially the similarities between archetype 1 and archetype 3 but also general similarities among all archetypes (eg, in the context of domain-specific, stand-alone CAs).

We found that much important information concerning the data processing of the CAs was missing in the publications. When information on data exchange with integrated third-party services or other things is missing, it is impossible to carefully judge the security risks. Therefore, we highly recommend that researchers exploit the taxonomy also as a reporting guideline when publishing on health CAs. Information on data privacy, security, and overall data processing is essential for generating trust in users, which might be important once such CAs become included in treatment workflows.

### Limitations and Further Research

Our research does not come without limitations. The classification of CAs from the literature required some interpretation as aspects such as hosting or data privacy were not clearly described. A unified terminology and a minimal data set of what should be reported on health CAs would help not only in grouping systems but also in judging their reliability. Therefore, the results depend on our understanding and interpretation of the literature base and, thus, possibly affect the internal validity. Although we have profound knowledge in the field of CAs, there is no guarantee of the “objective validity” of the k-means clusters. Consequently, the external validation of the clusters has to be a next step. Therefore, we highly encourage future research to challenge and enhance our classification system with different design characteristics.

Furthermore, the reviewed papers did not contain all the information required by our taxonomy. We were also interested in information on hosting, data privacy, or data exchange with third-party devices or services. We recognized that this information was missing in most publications (hosting 83/173, 48%, data privacy 157/173, 90.8%, and data exchange 120/173, 69.3%), although it is more important than ever for the successful transfer of CAs into actual practice [20]. Therefore, we ignored these characteristics in our clustering analysis. In addition, k-means clustering has some weaknesses (eg, we had to define the optimal number of clusters in advance, and the algorithm is sensitive to outliers). Future research should investigate the role and nature of objects that do not entirely fit into one of the clusters. Despite all limitations, we argue that our results reveal useful and highly valuable archetypes for health-related CAs, and to the best of our knowledge, this study is the first of its kind focusing on technical aspects.

For taxonomy building, we selected characteristics and dimensions that are of particular interest in health care settings to ensure data privacy and patient safety (eg, information on access to the internet, integration mode, and data privacy) and that also affect a technical evaluation. It is impossible to affirm the completeness of our taxonomy—technology is changing quickly, and it might become necessary to include additional characteristics or even dimensions. The taxonomy could also be used as a blueprint when designing a health intervention that exploits a CA. The taxonomy was generated in a pragmatic manner, and it is not yet validated. The number of categories remained the same during the process, but a few characteristics were added to the categories if needed during the annotation process. However, this procedure has no impact on the clustering as clustering was conducted after the manual annotation regarding our taxonomy was completed.

### Conclusions

In this paper, we presented a technical-oriented CA taxonomy for characterizing health CAs. A CA is considered from 4 perspectives: agent appearance, setting, interaction, and data processing. Using 13 relevant characteristics out of the 18 characteristics forming the taxonomy, 4 archetypes of health-related CAs were identified based on 173 unique CAs and using a k-means cluster analysis. We identified that the time dimension matters from a technical perspective to distinguish between archetypes. Moreover, we were able to identify additional distinctive, dominant, and nondominant characteristics that are relevant when evaluating health-related CAs (eg, input and output options or the complexity of the CA personality). Our archetypes reflect the current landscape of health CAs. With an increase in research interest in this field, which was already reflected in the literature review, we expect that more complex systems will arise. The archetype-building process would have to be repeated after some time to check whether new design archetypes come up.

The resulting archetypes can be used to specify a set of technical metrics for their evaluation from a technical perspective. A careful technical evaluation of a health CA should take place before a clinical trial is conducted or the CA is used in an uncontrolled setting. In a next step, we will combine the archetypes and our previous work on technical evaluation metrics for health CAs [45] to define a specific set of technical evaluation metrics for each of our 4 archetypes.

## Appendix

Multimedia Appendix 1 Data extraction table.

Multimedia Appendix 2 Results of the cluster analysis. Highlighted in gray are characteristics that contributed to >50% of the conversational agents assigned to this cluster. Not considered in the clustering are sentiment and all dimensions of data processing (internet access, hosting, access to third-party services, and data privacy).

## Abbreviations

AI: artificial intelligence
CA: conversational agent
VR: virtual reality
