# Supplementary material for: Developing a Technical-Oriented Taxonomy to Define Archetypes of Conversational Agents in Health Care: Literature Review and Cluster Analysis
Source: J Med Internet Res. 2023 Jan 30;25:e41583. doi: 10.2196/41583 (PMC9926340; doi:10.2196/41583)
Supplement: Multimedia Appendix 2 [file jmir_v25i1e41583_app2.docx]

|  |  |  |  |  |  |  |
| --- | --- | --- | --- | --- | --- | --- |
|  |  | Arche-type Name | **Text-based ad-hoc supporter (1)** | **Multi-lingual, hybrid ad-hoc supporter (2)** | **Hybrid, single language temporary advisor (3)** | **Embodied temporary advisor (4)** |
|  |  | N | 107 | 18 | 41 | 15 |
| **Agent appea-rance** | **Personality of CA** | Simple | 94.4% | 72.2% | 92.7% | 26.7% |
|  |  | Complex | 3.7% | 27.8% | 2.4% | 73.3% |
|  | **Embodiment** | No | 93.5% | 88.9% | 80.5% | 6.7% |
|  |  | Avatar | 6.5% | 11.1% | 19.5% | 40% |
|  |  | Physical | 0% | 0% | 0% | 53.3% |
|  | **Application technology** | virtual reality | 0.9% | 0% | 0% | 73.3% |
|  |  | augmented reality | 0% | 0% | 2.4% | 0% |
|  |  | normal | 95.3% | 100% | 97.6% | 20% |
|  |  | vocal | 1.9% | 0% | 0% | 0% |
|  | **Intelligence framework** | rule-based | 59.8% | 55.6% | 51.2% | 66.7% |
|  |  | self-learning | 35.5% | 44.4% | 46.3% | 33.3% |
|  |  |  |  |  |  |  |
| **Setting** | **Context** | general purpose | 11.2% | 11.1% | 7.3% | 13.3% |
|  |  | domain specific | 88.8% | 88.9% | 92.7% | 86.7% |
|  | **Service duration** | ad-hoc supporter | 50.5% | 38.9% | 43.9% | 26.7% |
|  |  | persistant companion | 15% | 27.8% | 9.8% | 26.7% |
|  |  | temporary advisor | 33.6% | 33.3% | 46.3% | 46.7% |
|  | **Human involvement** | Diad | 90.7% | 94.4% | 95.1% | 100% |
|  |  | Triad | 7.5% | 5.6% | 4.9% | 0% |
|  |  | Quadriad | 0.9% | 0% | 0% | 0% |
|  |  |  |  |  |  |  |
| **Interac-tion** | **Input mode** | Written | 90.7% | 44.4% | 31.7% | 6.7% |
|  |  | Spoken | 3.7% | 0% | 2.4% | 20% |
|  |  | Visual | 0.9% | 0% | 7.3% | 0% |
|  |  | Hybrid | 2.8% | 55.6% | 58.5% | 66.7% |
|  |  | Haptic | 0% | 0% | 0% | 6.7% |
|  | **Output mode** | Written | 92.5% | 44.4% | 0% | 0% |
|  |  | Spoken | 3.7% | 0% | 2.4% | 20% |
|  |  | Visual | 0% | 0% | 2.4% | 0% |
|  |  | Hybrid | 2.8% | 55.6% | 95.1% | 80% |
|  |  | Haptic | 0% | 0% | 0% | 0% |
|  | **Service channel** | Smart-phone embedded software | 44.9% | 66.7% | 48.8% | 13.3% |
|  |  | social media | 25.2% | 16.7% | 17.1% | 0% |
|  |  | website (web-based) | 15% | 16.7% | 29.3% | 80% |
|  |  | smart speaker | 1.9% | 0% | 0% | 0% |
|  | **Device** | PC | 6.5% | 0% | 22% | 53.3% |
|  |  | mobile device | 77.6% | 100% | 73.2% | 33.3% |
|  |  | Other | 2.8% | 0% | 4.9% | 13.3% |
|  |  | Both | 1.9% | 0% | 0% | 0% |
|  | **Language** | single language | 99.1% | 0% | 100% | 100% |
|  |  | multi language | 0% | 100% | 0% | 0% |
|  | **Integration mode** | stand-alone | 53.3% | 72.2% | 70.7% | 86.7% |
|  |  | part of a system | 33.6% | 27.8% | 26.8% | 6.7% |
